# The road to recovery: a synthesis of outcomes from ecosystem restoration in tropical and sub-tropical Asian forests

**DOI:** 10.1098/rstb.2021.0090

**Published:** 2023-01-02

**Authors:** Lindsay F. Banin, Elizabeth H. Raine, Lucy M. Rowland, Robin L. Chazdon, Stuart W. Smith, Nur Estya Binte Rahman, Adam Butler, Christopher Philipson, Grahame G. Applegate, E. Petter Axelsson, Sugeng Budiharta, Siew Chin Chua, Mark E. J. Cutler, Stephen Elliott, Elva Gemita, Elia Godoong, Laura L. B. Graham, Robin M. Hayward, Andy Hector, Ulrik Ilstedt, Joel Jensen, Srinivasan Kasinathan, Christopher J. Kettle, Daniel Lussetti, Benjapan Manohan, Colin Maycock, Kang Min Ngo, Michael J. O'Brien, Anand M. Osuri, Glen Reynolds, Yap Sauwai, Stefan Scheu, Mangarah Silalahi, Eleanor M. Slade, Tom Swinfield, David A. Wardle, Charlotte Wheeler, Kok Loong Yeong, David F. R. P. Burslem

**Affiliations:** ^1^ UK Centre for Ecology & Hydrology, Bush Estate, Penicuik, Midlothian EH26 0QB, UK; ^2^ Department of Geography, University of Exeter, Laver Building, North Park Road, Exeter EX4 4QE, UK; ^3^ Tropical Forests and People Research Centre, Forest Research Institute, University of Sunshine Coast, 90 Sippy Downs Drive, Sippy Downs, 4556, Queensland, Australia; ^4^ Asian School of Environment, Nanyang Technological University, 50 Nanyang Avenue, Singapore 639798, Singapore; ^5^ Ecology, Conservation and Zoonosis Research and Enterprise Group, School of Applied Sciences, University of Brighton, Brighton, BN2 4GJ, UK; ^6^ Biomathematics and Statistics Scotland, JCMB, The King's Buildings, Peter Guthrie Tait Road, Edinburgh EH9 3FD, UK; ^7^ Permian Global Research Limited, Savoy Hill House, 7–10 Savoy Hill, London WC2R 0BU, UK; ^8^ Department of Wildlife, Fish and Environmental Studies, Swedish University of Agricultural Sciences, Skogsmarksgränd, Umeå 907 36, Sweden; ^9^ Department of Forest Ecology and Management, Swedish University of Agricultural Sciences, Skogsmarksgränd, Umeå 907 36, Sweden; ^10^ Research Centre for Ecology and Ethnobiology, National Agency for Research and Innovation (BRIN), Jl. Raya Jakarta-Bogor KM. 46, Cibinong, Bogor, West Java 16911, Indonesia; ^11^ Department of Biological Sciences, National University of Singapore, Block S3 #05-01 16 Science Drive 4, Singapore 117558, Singapore; ^12^ School of Social Sciences, University of Dundee, Dundee DD1 4HN, UK; ^13^ Environmental Science Research Centre, Science Faculty and Forest Restoration Research Unit, Biology Department, Chiang Mai University, Chiang Mai, 50200, Thailand; ^14^ PT Restorasi Ekosistem Indonesia, Jl. Dadali No. 32, Bogor 16161, Indonesia; ^15^ Faculty of Tropical Forestry, Universiti Malaysia Sabah, Kota Kinabalu, Sabah 88400, Malaysia; ^16^ Borneo Orangutan Survival Foundation, BOSF Mawas Program, Palangka Raya, Central Kalimantan, 73111, Indonesia; ^17^ Biological and Environmental Sciences, University of Stirling, Stirling FK9 4LA, UK; ^18^ Department of Biology, University of Oxford, South Parks Road, Oxford OX1 3RB, UK; ^19^ Nature Conservation Foundation, 1311, ‘Amritha’, 12th Main, Vijayanagar 1st Stage, Mysuru, Karnataka 570 017, India; ^20^ Department of Environmental Systems Science, ETH Zürich, Universitätstrasse 16, Zürich 8092, Switzerland; ^21^ Bioversity International, Via di San Domenico, 00153 Rome, Italy; ^22^ Forever Sabah, Jalan Penampang, Kota Kinabalu, Sabah 88300, Malaysia; ^23^ Área de Biodiversidad y Conservación, Universidad Rey Juan Carlos, c/Tulipán s/n., E-28933 Móstoles, Madrid, 28933, Spain; ^24^ South East Asia Rainforest Research Partnership, Danum Valley Field Centre, PO Box 60282, Lahad Datu, Sabah 91112, Malaysia; ^25^ Conservation & Environmental Management Division, Yayasan Sabah Group, Kota Kinabalu, Sabah 88817, Malaysia; ^26^ J.F. Blumenbach Institute of Zoology and Anthropology, University of Göttingen, Untere Karspüle 2, Göttingen 37073, Germany; ^27^ Centre of Biodiversity and Sustainable Land Use, University of Göttingen, 37073 Göttingen, Germany; ^28^ Department of Zoology, University of Cambridge, Downing St, Cambridge CB2 3EJ, UK; ^29^ Centre for International Forestry Research (CIFOR), Jalan CIFOR, Bogor 16115, Indonesia; ^30^ Leverhulme Centre for Climate Change Mitigation, School of Biosciences, University of Sheffield, Alfred Denny Building, Western Bank, Sheffield S10 2TN, UK; ^31^ School of Biological Sciences, University of Aberdeen, St Machar Drive, Aberdeen, Scotland AB24 3UU, UK

**Keywords:** carbon, biodiversity, degradation, regeneration, tree planting, nature-based solutions

## Abstract

Current policy is driving renewed impetus to restore forests to return ecological function, protect species, sequester carbon and secure livelihoods. Here we assess the contribution of tree planting to ecosystem restoration in tropical and sub-tropical Asia; we synthesize evidence on mortality and growth of planted trees at 176 sites and assess structural and biodiversity recovery of co-located actively restored and naturally regenerating forest plots. Mean mortality of planted trees was 18% 1 year after planting, increasing to 44% after 5 years. Mortality varied strongly by site and was typically *ca* 20% higher in open areas than degraded forest, with height at planting positively affecting survival. Size-standardized growth rates were negatively related to species-level wood density in degraded forest and plantations enrichment settings. Based on community-level data from 11 landscapes, active restoration resulted in faster accumulation of tree basal area and structural properties were closer to old-growth reference sites, relative to natural regeneration, but tree species richness did not differ. High variability in outcomes across sites indicates that planting for restoration is potentially rewarding but risky and context-dependent. Restoration projects must prepare for and manage commonly occurring challenges and align with efforts to protect and reconnect remaining forest areas.

The abstract of this article is available in Bahasa Indonesia in the electronic supplementary material.

This article is part of the theme issue ‘Understanding forest landscape restoration: reinforcing scientific foundations for the UN Decade on Ecosystem Restoration’.

## 1. Introduction

Despite the critical role of equatorial forests in the global carbon cycle and biodiversity conservation, recent decades have seen extensive tropical deforestation and degradation, with losses driven largely by logging and agricultural expansion [1–3]. Human-modified forests and secondary growth forests now account for the majority of forest cover [4]. Forest restoration is intended to mitigate damage from anthropogenic impacts by reinstating tree cover where forests occurred naturally. The growing focus on nature-based solutions (NbS) to address the climate crisis [5] has resulted in ambitious, high-level commitments for forest restoration and tree planting; for example, the Bonn Challenge aims to restore 350 million hectares of deforested land by 2030 (https://www.bonnchallenge.org/), with some countries pledging in excess of 10% of their land area to forest restoration [6]. With careful consideration of local priorities, forest restoration in developing countries *could* offer a so-called ‘triple win’ of reducing biodiversity losses and supporting sustainable development while contributing to local and global mitigation of and adaptation to climatic change.

Despite ambitions for vastly scaling up restoration efforts, the evidence for the efficacy of forest restoration is heterogeneous. Outcomes of restoration activities can vary widely, suggesting implementation challenges or competition for other land-uses, and complicating prediction of the efficacy of future interventions [7–9]. In practice, there has been an over-emphasis on numbers of trees planted as a metric for forest restoration success, rather than managing, protecting and monitoring how these planted trees perform over longer timescales [10,11]. There is demand for an improved evidence-base of the long-term outcomes, the timeframes required and uncertainties around restoration, and the environmental factors and management practices that influence the growth and survival of planted trees. These knowledge advances will help to ensure that limited resources available for forest restoration are used optimally [12].

Restoration may target some aspects of ecosystem functioning (termed ecosystem restoration) or attempt to recover the functions and biotic assemblages existing in native reference forest ecosystems (ecological restoration; see electronic supplementary material, box S1). Our study focuses on ecosystem restoration of tropical and subtropical forests in Asia, where forests have been subject to logging at varying intensities, fragmentation and conversion to other land-uses, and where restoration has been undertaken to return structure and function of forests for the purposes of restoration and future harvesting. The most common intervention in forest restoration in tropical/subtropical Asia is the planting of nursery-grown saplings, which is often supplemented by other treatments such as weeding, cutting climbers, liberation thinning or planting of nurse plants (e.g. [13,14]). We focus on the outcomes of tree planting as a tool for restoration. Publications to date typically report on individual or a few sites, but syntheses of evidence are needed for improved predictions of the outcomes that can be expected from forest restoration (e.g. [8,15–17]).

Asian forests have several distinct ecological features that may present challenges for vegetation recovery. Large areas of tropical Asian forest are dominated by the single tree family Dipterocarpaceae [18,19] which has relatively short dispersal distances through gravity and gyration of winged fruits [20]. The clustered spatial structure of dipterocarp populations may limit the capacity of forests to regenerate naturally in parts of the landscape that are remote from a seed source, and where logging has removed a large proportion of the mature reproductive trees [20–22]. Inter-annual mast fruiting events also govern reproduction and the availability of seed for regeneration, as well as seedling stocks for reforestation [23,24]. Peat swamp forests are a distinctive variant of Southeast Asian lowland forests [25] that have been heavily degraded by drainage, timber extraction and clearance [26]. These activities have increased the susceptibility of degraded peat swamp forests to fire and flooding, resulting in a particularly challenging environment for restoration [27–29].

### (a) Potential environmental and biotic determinants of forest recovery

Forest stands often show regeneration problems after harvesting or agricultural abandonment, including a failure of some tree species to recruit, which results in a shift in species composition [30,31]. Forest structural changes associated with disturbance and degradation alter microclimatic conditions, resulting in increased exposure to solar radiation, reduced humidity and more extreme temperatures [32–35]. In a global synthesis, Crouzeilles *et al.* [36] found that forest restoration was more successful when previous disturbance was less intensive, which may be driven by the availability of propagules for recovery, the environmental barriers to recovery and ongoing human disturbances. Assessment of the response of planted individuals helps to disentangle the first cause from the latter two.

Microclimatic conditions, determined by vegetation structure, may interact with other broader scale environmental factors (e.g. rainfall seasonality, soil conditions, temperature, elevation) to exacerbate negative effects of disturbance. For instance, Qie *et al.* [37] found El Niño-driven tree mortality was higher at forest edges than in intact forest in Borneo. Forest recovery may also vary according to soil conditions and topography, with steep slopes, shallow soils and exposed ridges creating challenging conditions for regenerating stems [38].

Heterogeneous environmental conditions in disturbed ecosystems can affect the relative performance of different tree species [31,39]. In an Indonesian restoration site, Kardiman *et al.* [40] found that growth and survival rates of 38 planted tree species varied in response to microhabitat, suggesting that species choice and site–species matching are critical for the success of planting programmes. Plant functional traits provide a framework for predicting whether species are well-adapted to a specific environmental setting [41,42]. Wood density is considered a key functional trait on the acquisitive–conservative trait spectrum [43]. Higher community-average wood densities are typically found where dry seasons are more intense or in well-drained soil conditions, where high wood density may offer hydraulic safety [44–46]. Wood density was positively related to survival of trees planted into pasture in a restoration site in Australia and peat swamp forests in Asia [47,48] while greater allocation to rooting depth enhanced tree survival in a seasonally dry forest in Costa Rica [49]. The role of functional traits in explaining recovery and determining species-specific responses has not been widely explored in Asian forests and predictive site–species matching is hampered by a lack of trait data for most species [47]. The most widely available functional trait information is wood density [50] which presents a preliminary opportunity to explore relationships between species-level vital rates and traits.

The diversity of plantings may affect long-term performance through ‘insurance’ and ‘portfolio’ effects that arise when species differ in their responses to environmental variation in space and time [13,51,52]. Diversity at multiple levels, including functional and genetic diversity, is important in building resilient communities for the future [53,54] but comes with the practical challenge of developing silvicultural and horticultural knowledge for many species.

Assessments of restoration outcomes typically focus on survival and growth of planted trees at individual sites. These metrics are useful indicators of early barriers to restoration [55], but ultimately the longer-term goal of restoration is the structural and compositional recovery of the whole plant community and broader ecosystem. Syntheses of plot-based data have estimated the average above-ground carbon accumulation rate in moist tropical forests naturally regenerating after clearance as *ca* 4–5 Mg C ha^−1^ yr^−1^ depending on location and disturbance history [56]. Analyses to date have focused on secondary growth after clearing [57] and we have fewer estimates for actively restored forests (see electronic supplementary material, Box S1) or those recovering from varying degrees of degradation. A pan-tropical meta-analysis found that recovery of vegetation structure and biodiversity (plants and animals) had better outcomes in naturally regenerating forests than those under active restoration (defined broadly as assisted recovery of an ecosystem that has been degraded, damaged or destroyed) [58]. However, most sites in this analysis were not using different restoration methods in co-located plots and thus not often directly comparable [59]. Since tree planting is costly and upscaling is challenged by the need for supporting infrastructure, it is imperative to determine when and where tree planting is necessary to meet restoration goals [60].

Establishing a sound evidence-base to understand the capacity, limitations and risks of restoration practices in terms of effects on carbon accumulation and plant community diversity and structure will benefit forest restoration decision-making. Here, we synthesize evidence on forest recovery and the efficacy of tree planting as an intervention for ecosystem restoration in the tropical and sub-tropical forests of South and Southeast Asia. Using a large database of published and primary data, we specifically ask the following questions:

1. What are the observed rates of mortality for planted trees in ecosystem restoration of tropical and sub-tropical Asian forests?
2. How do rates of mortality and growth of planted trees vary according to the biophysical environment (elevation, temperature, rainfall, substrate type), habitat condition and biotic factors (taxa planted, species richness of plantings and species wood density)?
3. What are the differences in community-level basal area, above-ground carbon and tree species richness between forests actively restored via tree planting compared with neighbouring naturally regenerating forests, at the same time since disturbance?

## 2. Methods

### (a) Data compilation

**Figure 1.**
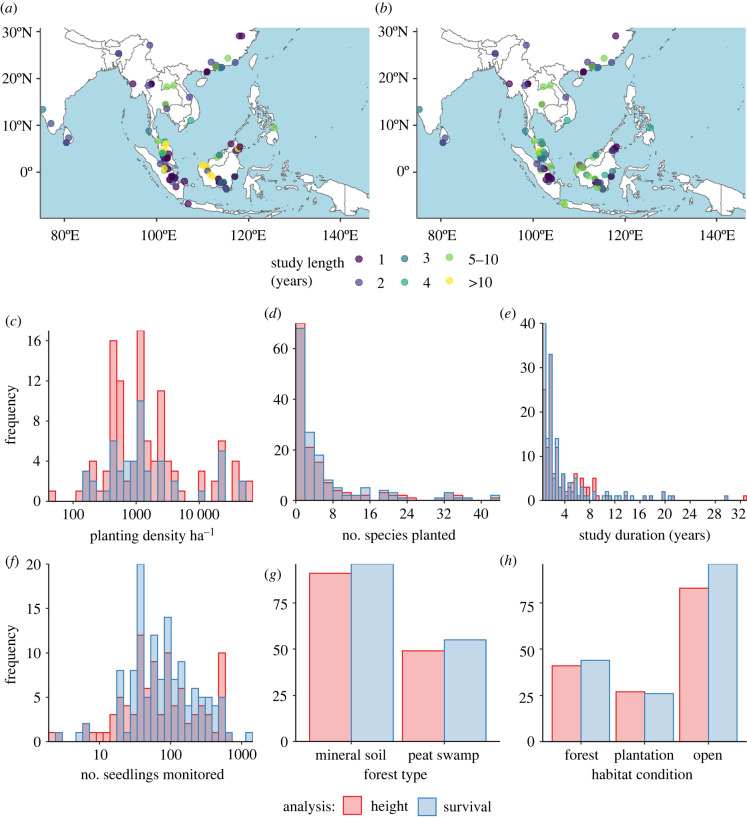
Maps of study locations and frequency distributions for variables in planted tree datasets. Map of study sites within the seedling (*a*) survival and (*b*) height datasets, showing study duration by colour (1 yr = 6 months to less than 18 months, 2 years = 18 months to less than 30 months, 3 years = 30 months to less than 42 months, 4 years = 42 months to less than 54 months, 5–10 years = 54 months to less than 114 months, greater than 10 years = 114–396 months). Frequency distributions (number of sites) are given for the height (red) and survival (blue) datasets for: (*c*) average planting density per site (ha^−1^); (*d*) average number of species planted in each treatment per site; (*e*) average length of study for each site; (*f*) average number of seedlings monitored per site; (*g*) number of sites in each forest type and (*h*) number of sites in each habitat condition category. (Online version in colour.)

We compiled data on planted tree survival, planted tree size and/or growth and area-based metrics of forest structure and diversity in planted and unplanted adjacent plots. Our datasets were compiled from online literature searches following the protocols outlined below (see electronic supplementary material, appendix S1 for further details), supplemented by additional published studies compiled by co-authors S.W.S. and N.E.B.R from peat swamp forest sites [47], and unpublished data and grey literature contributed by FOR-RESTOR network partners (www.ceh.ac.uk/our-science/projects/for-restor). We included forest restoration studies from tropical or subtropical, moist or dry broadleaf forest regions [61] of South and Southeast Asia (figure 1). We compiled additional studies from references cited in these papers, allowing us to capture regional and non-ISI indexed journals. We conducted our final bibliometric search on 15 May 2021 using the Web of Science database for studies in the English language. Some additional peat swamp forest studies were read in Indonesian by co-author N.E.B.R.

### (b) Planted tree-level data

We screened studies to assess whether they contained data on planted tree survival (numbers or proportions of trees that survived or died at known census intervals) and/or size or growth (measures of height, diameter, biomass or leaf number at multiple time points or calculations of growth that could be converted to size at each census). We read relevant studies in full and recorded numerical data or extracted them from figures using WebPlotDigitiser [62]. We were interested in trees planted in field conditions, excluding studies focusing only on naturally regenerating seedlings or those conducted in greenhouses or nurseries. We excluded studies where the final recorded census date was less than six months after planting to ensure timescales relevant for restoration. We recorded survival and growth metrics individually for each combination of site, species and treatment (where studies had replicated experimental treatments) for trees planted at the same time. We required there to be some information on disturbance history prior to planting and/or a statement on the purpose of the planting to ensure relevance to the question of ecosystem restoration (electronic supplementary material, table S2) and exclude studies only planting exotic commercial monocultures. A greater proportion of studies reported change in plant height than other measures of size, so we selected height as our metric for growth analyses.

For all relevant studies, we compiled data on ancillary variables to test for sources of variation in mortality and growth. We recorded the following information: location (country, latitude and longitude coordinates), duration of study, disturbance history type, site condition prior to planting, restoration methods, diversity of planting, planting density, seed source, tree age at planting and tree height at planting. We recorded diversity of planting and planting density at the treatment-within-species level, but for some studies it was necessary to apply site-wide average values. We categorized disturbance prior to restoration into the following classes: logging (clear fell and selective logging), agriculture (pasture, shifting cultivation, cultivation and grassland), plantation (oil palm, industrial monoculture), fire, mining or drainage based on the information provided on the most recent disturbance activity (usually the most severe). We classified the site condition at the time of planting into one of three categories: plantation enrichment (planting of native trees beneath non-native or monoculture plantations as defined above), natural forest enrichment (for secondary and logged forest sites) or open (with little or no tree cover), indicating whether trees were planted into existing tree canopy cover or more exposed conditions. We recorded six additional categories of restoration methods alongside planting (removal of competition, fertilizing, protection, shading, water regulation and soil preparation; see electronic supplementary material, appendix S1 for details). Due to the inconsistent reporting of site biophysical data (altitude, climate, soil), we extracted these variables from global datasets, as reported below.

We checked the taxonomic names given for the planted trees against global databases of known vascular plant species [63–65] and we unified synonyms by adopting the accepted scientific names given in World Flora Online [63]. In the few instances where there was uncertainty about the identification (e.g. trees were not identified at the genus level; species name was a synonym for multiple accepted scientific names; significant spelling errors), the data for that record were discarded. To test the role of functional traits in determining survival and growth, we extracted wood density data from the global wood density database [50,66,67] using the BIOMASS package in R [68]. Averages were calculated from globally distributed wood density values due to low sample sizes for some parts of Asia. From the 625 unique species included in our datasets, wood density values were assigned at the species (60.1%), genus (30.1%) or family level (8.8%) on the basis that wood density is known to be phylogenetically conserved in tropical Asia [69]. For five species, belonging to five families where family-level data were not available, the average of the entire dataset was used.

Overall, we compiled planted tree data from 176 restoration sites (221 199 trees) across South and Southeast Asia of which 148 sites (207 224 trees) reported data on survival and 136 sites (102 412 trees) reported height growth. This included 108 sites on mineral soils and 68 tropical peat swamp forest sites. In total, 625 tree species (252 genera; 81 families) were planted but species richness of plantings at individual sites was typically low (median of three, range = 1–49 species planted per site; figure 1). The five most common species planted were the dipterocarp species *Shorea balangeran* planted at 39 sites, followed by *Dyera polyphylla* in the Apocynaceae (31 sites), and the dipterocarps *Shorea leprosula* (26 sites), *Shorea parvifolia* (25 sites) and *Shorea ovalis* (16 sites). The five most common genera planted were the dipterocarps *Shorea* (88 sites), *Hopea* (35 sites), *Dipterocarpus* (21 sites) and *Dryobalanops* (20 sites) and *Dyera* (35 sites) (see electronic supplementary material, figure S1).

Although the maximum study duration was 33 years, only 49 (28%) sites conducted censuses 5 years or more after planting and the median study length was 2 years (figure 1). Median planting density was 1111 seedlings ha^−1^ (figure 1), but this information was only available for 42% of sites.

### (c) Environmental data

For each site, we extracted biophysical and climatic variables from the WorldClim datasets [70] at 2.5-minute resolution, averaged across years 1970 to 2000. Based on their potential impact on tree growth and survival we extracted elevation, mean annual temperature, mean annual precipitation and dry-season precipitation (precipitation in the driest consecutive three months). Mean annual precipitation and dry-season precipitation were highly correlated so we elected to proceed with dry-season precipitation. The Harmonized World Soil Database [71] misclassified soil at some peat swamp sites, and most forests on mineral soil had a similar classification, so our analyses proceed without using soil property data and instead use the dichotomous forest type classification of forests on mineral soil or peat swamp forest.

### (d) Community-level data

To establish differences in structure, biomass and biodiversity of naturally regenerating forest compared with sites where tree planting had taken place, we conducted a second search that focused on data available at the plot-level (electronic supplementary material, appendix S2). We screened 373 papers in total to identify studies that included monitoring of both natural regeneration and planted restoration plot(s) that were established within the same landscape at similar times and surveyed at least once for structural measures (basal area and/or above-ground biomass or carbon density) and indices of tree diversity. This resulted in 13 landscapes in total: data from 12 landscapes were extracted from 15 studies identified through the literature search, and co-authors provided data to derive additional metrics for two landscapes and one further unpublished landscape. The full dataset included a total area of 98.2 ha of restored forest across 452 plots (electronic supplementary material, table S5). Plots were surveyed from 0 to 50 years since disturbance and 0 to 30 years since planting took place.

The community-level analysis was carried out on the three metrics recorded from the literature search: above-ground carbon density, basal area and tree species richness. The means of comparison between planted and unplanted areas differed among studies: (i) 11 studies recorded plot-level metrics at one time point, allowing for comparisons between planted and unplanted plots; (ii) three studies recorded basal area at two or more time points allowing comparisons of change over time, and (iii) seven studies had recorded an old-growth reference value, against which both planted and unplanted plot-level metrics could be compared. As such, we conducted three statistical analyses, reported below, and the studies used in each of these analyses are indicated in electronic supplementary material, table S4.

### (e) Data analysis

#### (i) Planted tree mortality

To accommodate the heterogeneous census intervals among sites and studies and facilitate interpretation, we assessed mortality with separate models for each standardized time point (1, 2, 3, 4 years [±6 months], 5–10 years [54–114 months] and greater than 10 years [114–240 months] after planting). At each of the time points, we modelled mortality (number of dead and alive trees for a given site-species-treatment) via generalized linear mixed effects models with a binomial error distribution and a complementary log-log (cloglog) link function using the *glmmTMB* package [72]. To ascertain average mortality at each time point we fitted a model with an intercept term and random effects that accounted for variability associated with site, species and species-within-site (given that species may perform differently at different sites), and an observation-level random effect for overdispersion.

We then tested for the effects of environmental variables (climatic terms: mean annual temperature, dry-season precipitation, elevation), forest type, forest condition, species richness of plantings and wood density as additional fixed effects, with interaction terms between wood density and forest type and wood density and forest condition. We used the same random effects structure as with the intercept-only models. For models capturing studies over 5 years, we also tested the inclusion of a covariate to account for differences in ‘exposure to hazard’ (ln[time in months]). Continuous predictor variables (wood density, climate terms) were centred and scaled to facilitate model fitting. We used an information-theoretic approach to model selection, using the dredge function in the *MuMIn* package [73] which fits all combinations of fixed effects terms, while always retaining the specified random effects structure. We assessed the top-performing models (lowest AIC and with ΔAIC < 2). In those models we identified variables with importance value greater than 0.8 (which is the proportion of models within the top subset that contained that variables) and these variables were included in the final selected model [74]. In addition, we ran separate models to test for the effects of planting density and mean height at planting on mortality on tree mortality at 1 and 2 years after planting in open degraded and forest enrichment habitat classes.

We checked final models using the *DHARMa* package [75] and assessed the marginal effects of the fixed effects terms using the package *ggeffects* [76]. Model *R*^2^ values were calculated for fixed effects terms only (marginal *R*^2^) and fixed and random effects terms together (conditional *R*^2^) [77]. We examined the variation explained by each random effect through intra-class correlations (ICC), which indicate the strength of the correlation between data points within a group [77].

#### (ii) Annual size-standardized height growth rate

Height growth was censused across varying intervals (from 6 to 396 months) and with large variability in size at planting (mean = 42 cm, range = 2 cm–357 cm, figure 1). Since plant growth rate is known to be size-dependent [78–80] we chose to analyse annual size-standardized growth rate (AGR) rather than absolute growth. For each case (treatment-within-species and site) that had height recorded at four or more time points, we modelled height growth over time using linear or nonlinear least squares regression using three candidate functions (linear, exponential and Gompertz). When there were only three time points, only the linear and exponential fits were tested (further details in electronic supplementary material, appendix S3). We selected the regression model with the lowest AIC and visually checked model fits. We used the fitted curves to estimate the height at six months either side of the time at which trees reached a standardized height (namely, 100, 200 and 300 cm) and calculated the AGR as the difference between these two values (i.e. growth in 1 year when the tree is a given size; see electronic supplementary material, appendix S3 for details). The three standardized heights were chosen to capture a large proportion of our data without the need for extrapolating growth rates outside the range of the data.

AGR values were ln-transformed to meet the assumptions of a Gaussian regression model and modelled as response variables in linear mixed models using the *lme4* package [81]. The maximal models followed the same fixed effects structure as the mortality models. We included crossed random effects for site and species because variation explained by species-in-site was very minimal and caused problems with model fitting; likewise, the growth model for tree growth at 300 cm only included a random effect term for site. We used the same model selection and evaluation process as for mortality. Once the best model had been selected, we re-fit the model using a simulation approach to propagate uncertainty associated with the growth curve fitting stage of the analysis (detailed in electronic supplementary material, appendix S5). We found little difference between the two approaches in terms of parameter estimates and their confidence intervals and overall uncertainty contributed by the curve-fitting step was low (electronic supplementary material, tables S8 and S9) so our inference proceeded with the original models fitted to the observed data.

#### (iii) Community-level analysis

We analysed the three types of comparisons as follows. To compare naturally regenerating and actively restored plots we paired plots within a study based on the same time since disturbance; in the case of two studies [82,83] this matching meant we could not include data from all plots available. For each plot pairing we calculated a log-response ratio (equation (2.1)) that we term ‘restoration response’ for each metric, *m* (above-ground carbon density, basal area, tree species richness) which allowed us to unify comparisons where monitoring methods varied among sites (e.g. plot sizes, minimum tree size measured etc.) [84]. We tested the departure of the restoration response from zero for the three metrics by fitting a linear mixed effects model for each metric, where we included mean time since disturbance as a fixed effect, ‘study’ as a random factor and we weighted by total area surveyed in the plot pair.

2.1 $$response\;ratio\;{\left(restoration\;response\right)}_{m}=\ln\left(\frac{Active\;restoration{\;}_{m}}{Natural\;regeneration{\;}_{m}}\right)$$

To test for differences in basal area growth over time between the two restoration types (active restoration and naturally regeneration) we fit a linear mixed effects model with interacting fixed effects terms of time since disturbance and restoration type, with a random effect allowing the intercept and slope for each forest survey plot within a study to vary based on time since disturbance. We compared the AIC of the model with and without inclusion of the interaction effect and selected the best model based on the lowest AIC.

To compare the effect of restoration type (natural regeneration and active restoration) relative to undisturbed, reference forest for each of the three metrics, we calculated the ‘recovery completeness’ log-response ratio following Jones *et al.* [85] (equation (2.2)) for each metric, *m* (above-ground carbon density, basal area, tree species richness). We tested the difference in recovery completeness by fitting a linear mixed effects model, including time since disturbance and restoration type as fixed effects, ‘study’ as a random factor and we weighted by the area of the restoration plot. We compared the AIC with the model excluding each fixed effect and selected the best model based on the lowest AIC.

2.2 $$response\;ratio\;{\left(recovery\;completeness\right)}_{m}=\ln\left(\frac{Restored{\;}_{m}}{Old\;growth{\;}_{m}}\right)$$

All data analyses were undertaken in R v. 4.0.4 and 4.1.0 [86].

## 3. Results

### (a) Planted tree mortality

**Figure 2.**
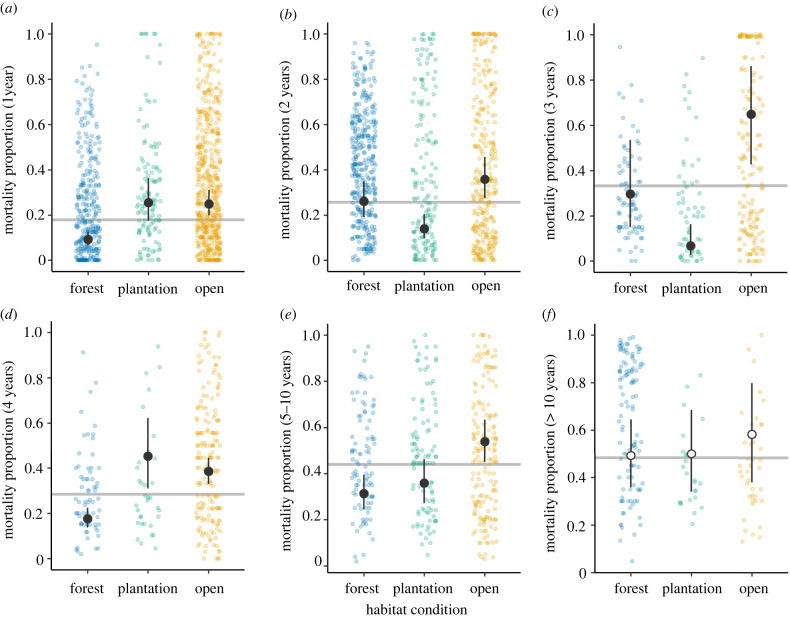
Mortality of planted trees is related to habitat condition. Coloured points are the observation level data (cases) where each point is a unique site–species–treatment combination at a given time point. Horizontal grey lines show overall mean mortality for that time point (95% CIs given in table 1) determined by the intercept-only models. (*a*–*e*) Black points and error bars show the estimated marginal mean mortality and 95% CIs for mortality by habitat condition class where tree planting was conducted (forest enrichment, plantation enrichment and open degraded habitats), as determined by the best GLMMs (see main text for details on model selection). (*f*) Habitat condition was not selected in the best model for cases greater than 10 years (table 1); white points (error bars) show the estimated marginal means (95% CIs) for mortality by habitat condition, with monitoring duration also included in the model. (Online version in colour.)

At 1 year after planting, average tree mortality was 18.0% (95% CI = 14.5–22.2%; figure 2 and table 1). Average mortality increased to 25.8% (95% CI = 20.1–32.7%) at 2 years and 44.0% (95% CI = 39.5–48.7%) mortality at 5–10 years after planting. Beyond 10 years, mortality was on average 48.3% (95% CI = 37.1–60.8%) (table 1). Mortality varied according to habitat condition and this term was selected in the top-performing models at all time points except for the longest running studies (greater than 10 years; *n*_sites_ = 14) (table 1). Mortality was consistently higher in open degraded sites than in forest enrichment sites (figure 2); at 1 year after planting, mean mortality was 9% (95% CI = 7–13%) in forest sites and 25% (95% CI = 20–31%) in open degraded sites. At 5–10 years after planting mortality was 31% (95% CI = 25–39%) in forest sites and 54% (95% CI = 45–63%) in open sites. In general, there were fewer observations from restoration within plantation sites. At some time points, mortality rates of plantings in plantations were more similar to forest enrichment settings (years 2, 3, 5–10) and at some time points more similar to open areas (years 1, 4).

**Table 1.** Testing drivers of seedling mortality. Sample sizes, mean mortality and 95% CIs estimated from intercept-only GLMMs for each time point. Results from GLMMs for each time point, where maximal models included environmental variables (mean annual temperature, dry-season precipitation, elevation), forest type, forest condition, species richness of plantings and wood density. Selected fixed effect terms are variables selected in best GLMMs (see electronic supplementary material, figures S3–S9 for bivariate relationships at each time point). Marginal *R*^2^, conditional *R*^2^ and intra-class correlations (ICC) showing variation explained by fixed and random effects terms.

no. of years post-planting	1	2	3	4	5 to 10	>10
sample size:						
*n* cases	973	828	304	283	375	179
*n* sites	93	66	33	20	26	14
*n* species	327	303	159	131	186	70
mean proportional mortality	0.18	0.258	0.333	0.285	0.44	0.483
(95% confidence intervals)	(0.145–0.222)	(0.201–0.327)	(0.211–0.500)	(0.226–0.355)	(0.395–0.487)	(0.371–0.608)
selected fixed effects terms	habitat condition; forest type; elevation	habitat condition	habitat condition; species richness	habitat condition; dry season precipitation; temperature	habitat condition; forest type	monitoring duration
marginal *R*^2^	0.104	0.039	0.239	0.07	0.037	0.02
conditional *R*^2^	0.418	0.481	0.648	0.282	0.302	0.227
ICC:						
site	0.246	0.322	0.47	<0.001	0.049	0.116
species	0.038	0.041	0.067	0.042	0.059	0.031
species in site	0.067	0.097	<0.001	0.185	0.168	0.064
observation level	0.179	0.086	0.097	0.102	0.051	0.038

Mortality was higher in peat swamp forests (28% [95% CI = 20–39%]) than in forests on mineral soil (14% [95% CI = 11–19%]) at 1 year after planting but not in subsequent years (electronic supplementary material, appendix S4; figure S3). At 5–10 years after planting, forest type was also selected in the best model, with mean mortality higher in forests on mineral soil (44% [95% CI = 37–51%]) than peat swamp forest (30% [95% CI = 19–45%]) but the confidence intervals were overlapping and sample size was low for peat swamp forests (*n*_sites_ = 5, *n*_cases_ = 35). Peat swamp sites were more frequently classed as open degraded than the other habitat classes; we checked the influence of peat swamp sites on our results and found the same effect of habitat condition on mortality on mineral soils alone as with the full dataset.

Broadly, the other covariates we tested (elevation, climatic terms, planted species richness and wood density) were not important drivers of mortality in our models. In several instances, additional variables were selected in the best models—at 1 year after planting, elevation was positively associated with mortality rates; at 4 years after planting, temperature was negatively associated and dry-season precipitation positively associated with mortality (table 1). However, the explanatory power of the fixed effects was low (marginal *R*^2^ ranging from 2 to 24% of variation explained across models, table 1) and confidence intervals broad (electronic supplementary material, figures S4–S8).

A greater proportion of variation was captured by the random effects terms (conditional *R*^2^ in table 1, ranging from 23% to 65%). In the first 3 years after planting, the site term explained the greatest variation (25–47%) (ICC; table 1); at 4 years after planting, inter-site variation was partially accounted for by fixed effect terms temperature and dry-season precipitation. Variation explained by species across the dataset was generally lower (less than 7%), with a higher correlation between observations of the same species within sites (table 1).

We found no effect of planting density on mortality (at 1 and 2 years after planting, in either open degraded or forest enrichment settings). However, greater height at planting reduced mortality rates in open degraded habitats at year 1 and marginally at year 2 (electronic supplementary material, appendix S4, figure S9) while in forest enrichment habitats height was negatively associated with mortality in year 1 but they were not related at year 2.

### (b) Annual growth rates

**Figure 3.**
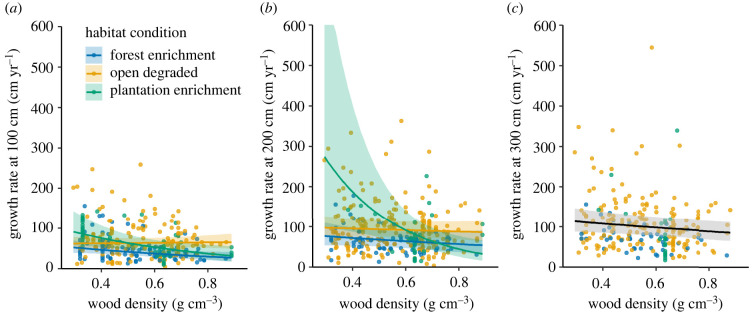
Size-standardized annual height growth rate is related to wood density and habitat condition. The panels show the variables selected in the best models for growth rate at 100, 200 and 300 cm. Each point (case) gives the estimated growth rate for a given site, species and treatment. Coloured lines give predictions with shaded 95% confidence intervals (truncated to 600 cm yr^−1^ in panel *b*) when habitat condition was significant in the models. At 300 cm, habitat condition was not significant and the mean relationship between growth and wood density is shown (black line with shaded 95% CIs). (Online version in colour.)

Wood density (g cm^−^^3^) was generally negatively associated with tree growth rate (cm yr^−1^) at all three reference sizes (100, 200 and 300 cm), but the effect of wood density on growth varied according to habitat condition at the first two reference sizes. There was no effect of wood density on growth in open degraded conditions, but these terms were negatively related in forest and plantation enrichment environments (figure 3*a,b*; electronic supplementary material, table S9). At 100 cm and average wood density (0.54 g cm^−3^), growth rate was higher in open conditions (63.8 [95% CI = 54.1–75.4] cm yr^−1^) and plantations (59.0 [95% CI = 44.6–78.0] cm yr^−1^) than in degraded forest (40.0 [95% CI = 31.8–50.3] cm yr^−1^). Similarly, at 200 cm and average wood density (0.56 g cm^−3^), growth rate was higher in open conditions (92.5 [95% CI=74.9–114.3] cm yr^-1^) and plantations (107 [95% CI = 66.4–172.8] cm yr^−1^) than in degraded forest (65.9 [95% CI = 48.8–89.0] cm yr^−1^). There was no significant effect of habitat condition at 300 cm reference size which may indicate that this effect is size-dependent, but the number of sites with trees of this reference size was also fewer (*n* = 25; table 2).

**Table 2.** Testing drivers of seedling growth. Summaries of best linear mixed effects models for ln(growth rate (cm yr^−1^)) at three reference sizes: 100 cm, 200 cm and 300 cm. Maximal models included environmental variables (mean annual temperature, dry-season precipitation, elevation), forest type, forest condition, species richness of plantings and wood density. Selected fixed effect terms are variables selected in best LMMs. Marginal *R*^2^, conditional *R*^2^ and intra-class correlations (ICC) showing variation explained by fixed and random effects terms.

seedling reference size (cm)	100	200	300
sample size:			
*n* cases	310	341	256
*n* sites	50	38	25
*n* species	163	180	144
selected fixed effects terms	habitat condition; wood density; condition: wood density interaction	habitat condition; wood density; condition: wood density interaction	wood density
marginal *R*^2^	0.145	0.057	0.009
conditional *R*^2^	0.547	0.593	0.657
ICC:			
site	0.384	0.434	0.654
species	0.086	0.134	NA

We did not detect any difference in growth rates between peat swamp forests and forests on mineral soils. None of the other environmental variables tested had an effect on growth, and in general the marginal *R*^2^ values were low (0.145, 0.057 and 0.009 for the models for 100 cm, 200 cm and 300 cm reference sizes respectively; table 2). As with the mortality rates, more variation in growth was explained by the random effects terms (see conditional *R*^2^, table 2). The site-level random effect explained 38.4–65.4% of variation in growth rate across the models for the three size classes (see ICCs, table 2). Species (across the whole dataset) accounted for 8.6–13.4% of variation in growth at 100 cm and 200 cm respectively; it was not possible to calculate variation explained by species within site.

### (c) Community-level studies

**Figure 4.**
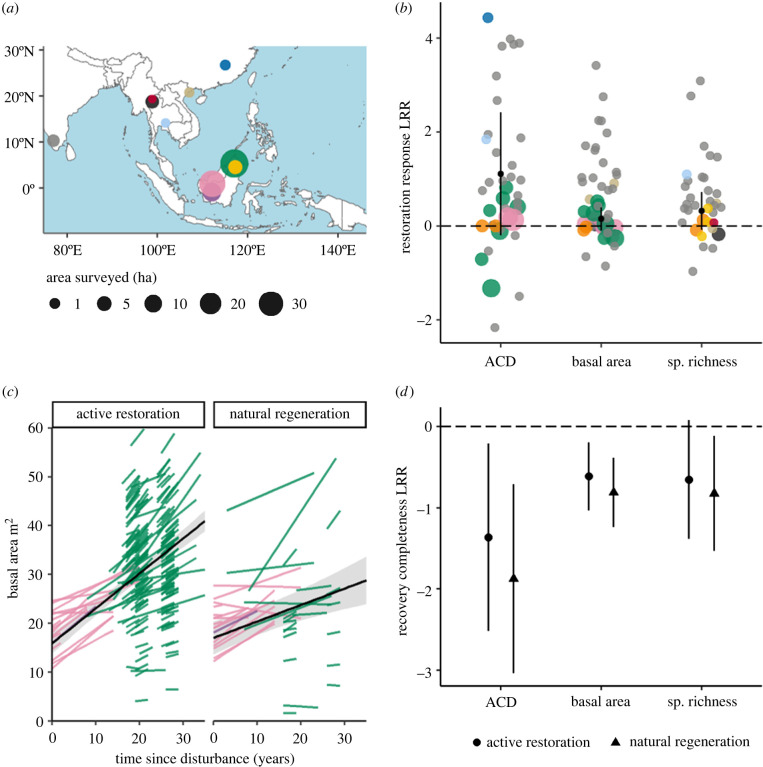
Community-level responses to active restoration by tree planting. (*a*) Map of studies included in community-level analyses, with size of point representing total area of plots surveyed in the study. Study colours correspond with those used in analyses for (*b*) and (*c*). (*b*) Restoration response (log-response ratio; LRR, equation (2.1)) for structure (above-ground carbon density, (ACD; Mg C ha^−1^); basal area (m^2^ ha^−1^)) and diversity (tree species richness) metrics—each point is a paired comparison between active restored and naturally regenerating plots within a study, where point size represents area of plots within pairs. Black points are mean restoration response with 95% confidence intervals. (*c*) Mean predictions (black lines with 95% confidence interval shaded) and plot-level random effects estimates (coloured by study) for relationships between basal area and time for actively restored and naturally regenerating plots. (*d*) Recovery completeness (log-response ratio; LRR, equation (2.2)) for structure and diversity metrics—each point is the mean recovery completeness across studies (where a value closer to zero represents metric value closer to old-growth reference), with 95% confidence intervals. (Online version in colour.)

When we compared actively restored (planted) plots with naturally regenerating plots we found no significant effect of planting on above-ground carbon density (restoration response = 1.108, 95% CI = −0.199–2.414 at average time since disturbance = 20.2 years, *n*_studies_ = 6, *n*_plot pairings_ = 38), tree species richness (restoration response = 0.321, 95% CI = −0.078–0.720 at average time since disturbance = 20.6 years, *n*_studies_ = 6, *n*_plot pairings_ = 35) or basal area (restoration response = 0.149, 95% CI = −0.093–0.391, at average time since disturbance = 19 years, *n*_studies_ = 7, *n*_plot pairings_ = 43) (figure 4*b*, electronic supplementary material, appendix S6). While, on average, above-ground carbon density, basal area and tree species richness were higher in actively restored plots than in naturally regenerating plots, in all three metrics the 95% CI of the restoration response overlapped zero (figure 4*b*).

When we modelled basal area change over time, we found that on average actively restored plots accumulated basal area more quickly (0.715 m^2^ yr^−1^, 95% CI = 0.617–0.813 m^2^ yr^−1^) than nearby naturally regenerating plots (0.336 m^2^ yr^−1^, 95% CI = 0.136–0.436 m^2^ yr^−1^), as indicated by a significant interaction between time since disturbance and restoration type (ΔAIC = 11.5, *n*_studies_ = 3, *n*_plots_ = 287; figure 4*c*).

The above-ground carbon density and basal area of actively restored plots were significantly closer to reference values for old growth forests than naturally regenerating plots (the model including a term for restoration type had the lowest AIC), but there was no significant difference between actively restored and naturally regenerating plots in terms of tree species richness (figure 4*d*). Despite having more similar metric values, actively restored plots were still significantly lower than old-growth forests, in terms of above-ground carbon density (recovery completeness = −1.363, [95% CI = −2.520–−0.206], at average time since disturbance = 18.7 years) and basal area (recovery completeness = −0.612 [95% CI = −1.033–−0.192], at average time since disturbance = 18.2 years) but tree species richness of old growth forests fell within the 95% confidence interval of actively restored plots (recovery completeness = −0.652 [95% CI = −1.383–0.080], average time since disturbance = 23.7 years).

Across the three analyses, there was a tendency for active restoration to have a positive effect on basal area and above-ground carbon density recovery relative to natural regeneration, but results were sensitive to the type of analysis and combination of sites.

## 4. Discussion

### (a) Demographic fate of planted trees

Our synthesis revealed that, on average, *ca* 20% of planted trees died within 1 year and *ca* 50% had died once plantings were beyond 10 years old. However, there was a large amount of variability around these mean mortality rates. Broad-scale climatic gradients explained very little of this variation, but mortality was highly correlated at the site level indicating there are environmental, methodological or social factors operating which affect whole sites and that restoration outcomes are highly context dependent.

Mortality was consistently lower at forest enrichment sites than in open environments, supporting previous global and regional studies which also found habitat condition to be an important driver of outcomes. Crouzeilles *et al.* [36] found that, globally, forest restoration was more successful when previous disturbance was less intensive, and in a review of enrichment planting, Paquette *et al.* [30] found that survival of under-planted trees increased, up to a threshold, with increasing shade-tree cover. Transplanted seed and seedling survival were highest in older secondary forest versus young forest across a restoration chronosequence in tropical Australia [87]. Enhanced seedling survival under tree canopies may result from the amelioration of extremes in light, temperature and water availability which could be particularly problematic at early stages of establishment. Provision of shade may be important for certain tree species (e.g. some dipterocarps) which lack tolerance of high light and temperature [88,89]. Qie *et al.* [31] found naturally regenerating seedlings had species-specific responses to levels of forest degradation in Borneo, which is supported by evidence showing that some species have a capacity to acclimate to conditions in newly logged forests [90].

Proximity to established trees may have several other ecosystem benefits. An established tree canopy facilitates rapid colonization of planted seedlings’ root systems by mutualistic fungi, which are known to enhance seedling survival and growth [91], whereas soil physical, chemical and biological properties may be more disturbed in open sites as a result of their disturbance history. This could be particularly important in Asian dipterocarp forests because dipterocarps form obligate ectomycorrhizal associations [19,91–93]. Lower rates of survival in open degraded forests may also result from increased competition and suppression by native and non-native grasses, weeds, ferns and climbers; the presence of grasses was found to be one of the leading factors in determining natural regeneration outcomes in a Costa Rican landscape [94]. Overall, existing trees in the landscape support restoration by enhancing the survival of planted trees, for example, in or adjacent to degraded forest fragments; protecting forest remnants is vital to restoration and the creation of forest ‘islands’ (applied nucleation) may play a similar function [95,96].

Tree mortality rates were higher in peat swamp forest than forest on mineral soil in the first year after planting, potentially reflecting additional environmental challenges posed in degraded peatlands including higher susceptibility to fire, peat subsidence and water table fluctuations following drainage [27,97]. However, a recent review of peat swamp studies showed no significant effect of prior drainage on seedling mortality, but found species choices to be important—slower growing tree species survived for longer [47]. In our study, there were fewer peat swamp sites monitored over longer time periods, but a better ability to detect ongoing differences would help to elucidate drivers of differing outcomes between habitats. Predicted tree mortality in plantations was variable across time points, which may also have been driven by the low number of sites and the inherent variability among those that were available for analysis. The higher mortality rates in open degraded and peat swamp sites indicate that the future of plantings is more uncertain in these habitats, yet these sites may also be the least likely to recover spontaneously because of the low residual density of large adult trees and challenging environments in these settings. More intensive management and maintenance may reduce these risks and improve outcomes.

Few studies gave a quantitative record of baseline conditions—this information would help to determine thresholds at which certain risks or outcomes may occur and guide restoration planning [12,98,99]. Risks may be mitigated, through more intense maintenance and/or appropriate seedling stocking densities to bring about rapid canopy closure in very degraded systems [94,98]. We found a positive effect of tree height at planting on survival in the open degraded sites, as has been found in other studies [100]. Larger seedlings are usually better equipped to withstand environmental challenges or maximize on opportunities for rapid growth, but they are also more costly to produce and plant so the cost−benefit trade-off must be carefully assessed.

### (b) Composition, diversity and wood density of plantings

A large variety of tree species were used in restoration plantings across Asia (a minimum of 625 species) but, on average, species richness at any one site was low (median = 3 species), indicating that only a small fraction of the native species pool is used in restoration. This may reflect challenges associated with seedling supply [101], particularly of rare species, and the uncertainties over suitable propagation and silvicultural techniques for many species. Variability in mortality rates may arise because of species-specific responses and the appropriateness of selected species to the planting environment. Yet, we found that typically the effect of site was stronger than that of species. Partitioning individual species level effects is difficult when planting diversity is low and when higher-level drivers of mortality are affecting all species, but it could also be that some projects selected a few, well-known ‘safe’ species with broad tolerances. In Asian forests, species assemblages are often strongly habitat-associated and soil environments are heterogeneous [102–104]. This may pose challenges for restoration in the region because species may perform very differently over small spatial scales.

We did not find a strong effect of the number of planted species on survival, although detecting the effects of richness on vital rates was hampered by the low species richness of plantings at most sites. The effect of planted species diversity will also depend on the diversity of the baseline community pre-planting and the spatial arrangement of planting (i.e. the local mixtures), for which we lacked data. More broadly, the effects of low diversity in plantings may play out over time spans longer than we were able to investigate, driven by increasing susceptibility to plant diseases [52] or reduced complementarity of space-filling and resource use among the planted cohort [13,105]. A positive relationship between diversity and survival was observed in a large-scale experiment included in our synthesis after 10 years [13]. Bongers *et al.* [106] showed an increasing importance of diversity on forest productivity with time, which emphasizes the need for long-term monitoring in restoration settings.

Wood density was not an important determinant of tree mortality in our synthesis; this contrasted with a study of seedling survival in seasonally dry forest in Australia where Charles *et al.* [48] found a positive relationship between wood density and survival of seedlings in restored rainforest. Relationships between wood density and vital rates are likely to be nuanced and context dependent [43]. In our study, wood density displayed a negative relationship with height growth in degraded forest and plantation settings at smaller plant sizes (100 cm and 200 cm) where it may be particularly beneficial to allocate carbon to growth as opposed to wood density. Lack of competition from neighbouring trees and a high light environment may mean wood density is a less important driver of growth in open ecosystems [107,108]. A recent synthesis [100] found that in humid forests, acquisitive traits (high specific leaf area, low wood density) maximized the positive effect of seedling size on tree seedling survival in a restoration context, and achieving rapid canopy closure may help to provide the environment for recovery of species more sensitive to climatic stress [109,110]. We were only able to examine wood density as a measure of plant function, while other traits may be important in influencing survival, growth and their trade-offs. Furthermore, we lacked finer-resolution soil information which is likely to be important for contextualizing trait–rate relationships. Building further understanding in this area may help guide species choices when planning for restoration outcomes [111].

### (c) Biases and gaps in our understanding of restoration outcomes

The monitoring of over 200 000 seedlings represents a vast logistical effort by the original research teams, and the synthesis has allowed us to identify some high-level systematic effects. Nonetheless, our synthesis may suffer from several sources of bias. Firstly, we observed relatively low average mortality in the first 2 years after planting—closely monitored sites with the intention of reporting or scientific publishing may have had more tightly controlled conditions which might not apply in large-scale reforestation projects or those with less thorough monitoring. A recent review revealed that while organizations engaged in tree planting have increased by 288% in the last three decades, only 5% of project websites and reports mentioned monitoring [11]. Furthermore, only sites with sufficiently high survival will continue to be monitored over longer timescales—this combined with often-limited financial support for long-term remeasurement means that sample sizes of sites measured for over 10 years are low and almost certainly biased. While some data from unpublished sources and the grey literature were included in our database, evidence assembled from published literature may be biased in favour of sites displaying higher survival. Two studies in our synthesis reported survival rates after catastrophic fires [112,113], while one experimental site within the FOR-RESTOR network suffered significant mortality after the survival rates were published [40]. A few studies did report incidences where species or sites were not measured because they displayed unusually high mortality rates, for example, due to disturbance by animals [114,115] weeds [116], or unknown/unreported causes [117–120]. Ultimately, we expect our estimates are biased and perhaps better reflect *restoration potential* than a realistic average of all restoration projects being undertaken across a range of sectors.

Our study largely focuses on the environmental and ecological controls on restoration outcomes, but governance, tenure and land-use likely contribute to the differences we observed among sites, and perhaps systematically so among habitat condition classes. Areas with an existing forest canopy are more likely to be owned or managed by a state forest service, and be remote from human settlements, than areas that lack a tree canopy. Forested areas may suffer less from encroaching domestic animals and spill-over fires from cultivated land than areas that are fully deforested; conversely local communities can form networks to protect land. Restoration practitioners more frequently reported social factors as important to restoration longevity that ecological factors [121] but these contextual factors are less frequently reported.

### (d) Community-level restoration outcomes

Data on whole-community responses were spatially sparse, covering just 11 landscapes and *ca* 90 ha in area, with studies taking different approaches to assessing restoration impacts. The findings indicate that active restoration through tree planting can be beneficial to increasing the speed at which basal area recovers in degraded forests (figure 4*c*) and in approaching the biomass and basal area found in neighbouring old growth forests (figure 4*d*). This supports the findings published by [82] who found that actively restored forests in Sabah gained 50% more above-ground carbon per hectare per year than naturally regenerating forests over the course of a decade. Despite these findings, we did not detect a significant difference between the basal area and above-ground carbon of actively restored and naturally regenerating forests directly; we attribute this to the fact all analyses have relatively low sample sizes and are sensitive to the particular mixture of sites, indicating the need for more evidence. Added to this, restoration projects are not often established as strict ‘experiments’ and thus there may be factors such as level of degradation and level of intervention that are not true paired comparisons, making it difficult to partition the effect of restoration treatment.

The differences in above-ground carbon accumulation and basal area increment we found between planted and naturally regenerating areas were less pronounced than differences in wood volume change presented by Shoo *et al.* [122] in Australia, possibly because the Australian sites were recovering from clearance whereas many sites in our comparison were regenerating after selective logging (eight of 11 landscapes). The effects of active restoration in degraded forests (when compared with open areas) may be more subtle because natural regeneration is supported in untreated areas by remaining mature trees. We did not have the power to test for habitat condition effects but we did observe variation between studies within our own dataset, for example, the plots in Western Ghats [123] had especially positive responses to active restoration and we hypothesize this may result from the sites being in isolated fragments in an agricultural setting with the invasive shrub *Lantana camara* present*;* both of these factors may be reducing natural regeneration in the degraded forest, meaning that active restoration has a disproportionate effect on forest recovery [96].

Our synthesis focused on the role of tree planting in delivering biomass and tree biodiversity recovery, driven by the availability of comparable data. Other interventions may be effective in accelerating forest recovery, particularly in less disturbed forests, for example, weed and climber cutting [124,125] and thinning of dominant or early successional species [126,127], applied alone or in combination with planting. In some of the studies we compiled, tree planting was undertaken concurrently with other restoration activities; it is therefore difficult to tease apart which treatment had the greatest effect, or the ways in which they may be complementary, creating conditions conducive to germination and tree-seedling establishment. Controlled experiments that compare restoration techniques are needed, implemented over a wide range of forest types and environmental conditions, to identify the circumstances under which each intervention method would be most effective (e.g. [15,59]). While we did not detect strong effects of restoration on tree species richness, we advocate further research on changes in species composition and distributional or functional attributes of species that recover or fail to recruit under different restoration treatments.

The ability to look in greater depth at differences between silvicultural treatments through time would be beneficial (e.g. [122])—are differences in structure and biodiversity related to restoration approach maintained over long timescales or do naturally regenerating sites ‘catch up’? Evidence suggests that naturally regenerating forests can recover their structure after about six decades [128,129] but there are risks to natural regeneration if ecological or socio-political barriers persist or protection is lacking [130,131]. Landscape-level factors (e.g. distances to natural forest remnants) are likely to be influential in determining the extent to which natural regeneration is viable [36,132].

## 5. Conclusion and recommendations

Our synthesis has identified that: (i) the outcomes of tree planting are highly variable, but planting is a comparatively costly approach to restoration, so we must improve the understanding of ‘permanence drivers’ and assess the cost–benefits of different restoration interventions in different landscape contexts. Ecological and social drivers of success are typically studied in isolation, while in reality an integrated assessment of the socioecological context is required to inform restoration outcomes [133]. The uncertainty in planting outcomes also emphasizes the critical value of protecting remaining functionally intact forests. (ii) Restoration outcomes are context-dependent, related to site conditions and species choices. Improved capture of quantitative data on baseline environmental and vegetation conditions (including competing floristic elements) and regular measurements will help elucidate drivers of restoration success more thoroughly. This requires valuing, involving and strengthening local knowledge. (iii) Species richness of plantings were generally low, which may affect future forest function. Barriers to incorporating greater diversity in plantings should be explored and addressed. (iv) Our synthesis showed that average seedling mortality increased by *ca* 30% between 2 and 5–10 years post-planting, but few sites were monitored to this point. Greater financial and institutional support for long-term monitoring and reporting of large-scale mortality events and their causes (environmental and anthropogenic) are needed [133]. (v) The main evidence gaps for tropical and subtropical Asia concern the relative success of natural regeneration, responses to restoration interventions at the scale of whole community and data from experiments with well-designed controls. This new research should address the relative roles of different restoration methods, how trajectories evolve over time and how different taxa may be affected. To improve our collective understanding of restoration outcomes, we advocate improved practices and institutional and financial support for collecting and sharing restoration evidence [134]. We hope to contribute to this with our growing FOR-RESTOR network (www.ceh.ac.uk/our-science/projects/for-restor), an Asian regional hub for collaboration, information and data sharing, for archiving experience and disseminating good practice.

## Data Availability

Data and code for the statistical models are made available through the UKCEH Environmental Information Data Centre (EIDC). Supplementary material is available online [135].
